# Factors associated with lumbar disc high-intensity zone (HIZ) on T2-weighted magnetic resonance image: a retrospective study of 3185 discs in 637 patients

**DOI:** 10.1186/s13018-018-1010-z

**Published:** 2018-12-04

**Authors:** Zi-Xuan Wang, You-Gu Hu

**Affiliations:** 1 Department of Interventional Radiology, Qingdao Municipal Hospital, Qingdao, 266000 Shandong People’s Republic of China; 2 Department of Spinal Surgery, Affiliated Hospital of Qingdao University, Qingdao, 266003 Shandong People’s Republic of China

**Keywords:** Intervertebral disc, MRI, High-intensity zone, Low back pain

## Abstract

**Background:**

It is well known that internal disc disruption (IDD) is accelerated by factors such as aging and injury. High- intensity zone (HIZ) on lumbar MRI is usually considered a marker of painful IDD. However, many painful IDD show no HIZ. This suggests that the risk factors of HIZ may be different to these of IDD. The purpose was to clarify the correlation between the HIZ on lumbar MR and the factors, including gender, age, body weight, and low back pain (LBP).

**Methods:**

Characteristics were obtained from the medical record. The MR images, biplanar post-discography radiographs, and post-discography CT images were reviewed and rated by two experienced radiologists in a blinded fashion.

**Results:**

Annular HIZ correlated significantly with age (OR = 1.011), body weight (OR = 1.022), and LBP symptom (OR = 1.527). The lowest two HIZ prevalence rates were in the second and the third decades (11.54% and 7.84%). The highest prevalence was in the sixth decade (38.03%). The body weight was positively associated with the HIZ prevalence. There was a significant difference in HIZ prevalence between symptomatic and asymptomatic patients (36.16% vs. 26.96%, *P* < 0.05). All the HIZ discs exhibited grade 3 or grade 4 disruptions, but only 9 discs (9/16, 8 exhibited grade 4 annular tears) were detected with exact pain reproduction.

**Conclusions:**

It is demonstrated that the presence of HIZ on lumbar MR image was associated with aging, high body weight, and low back pain symptom. HIZ sign indicated a part of the natural history of disc degeneration but was not an actual source of low back pain.

## Background

Discogenic low back pain (LBP) is a symptom of internal disc disruption (IDD) [1]. This condition is characterized by disruption of the internal architecture of the disc, which can be demonstrated by computed tomography (CT)/discography [2]. In 1992, Aprill and Bogduk [3] reported a diagnostic sign of painful lumbar disc, high- intensity zone (HIZ), on T2-weighted magnetic resonance (MR) images and considered that the HIZ, when present, is pathognomonic of an internally annulus disrupted and symptomatic intervertebral disc. HIZ is analogous to the radial fissures extending from a nucleus pulposus to the outer annulus as described by Ross et al. [4], which enhanced on injection of gadolinium-DTPA, a behavior taken to indicate inflammation, neovascularization, and which has been shown clinically to correspond to granulation tissue in HIZ disc taken from disc operation by Peng and Hou et al. [5]. In the following decades, some other studies revealed the correlation between HIZ sign and symptomatic disc with discography [5–13]. It is believed that the HIZ is a marker of painful IDD.

Nowadays, the use of magnetic resonance image becomes widespread in clinical practice. Therefore, we are concerned that the number of HIZs founded on MR images will increase. Accordingly, we need to clarify which acquired factors promote HIZ so as to reveal the risk factors of HIZ and establish preventive measures against HIZ and related symptomatic IDD.

It is well known that IDD is accelerated by several factors such as aging and injury [14]. However, most IDD patients show no HIZ on MR images. This suggests that the risk factors of HIZ may be different to these of IDD. Therefore, what are the factors associated with HIZ and what are the features of these risk factors? From the literature, a number of studies focusing on the significance of HIZ in symptomatic and/or asymptomatic patients have been well documented [5–13]. Nevertheless, few researches are performed to explore the risk factors related to HIZ sign. In order to clarify the correlation between gender, age, body weight, low back pain, and HIZ sign, we constructed this retrospectively study and reviewed 3185 discs in 637 patients on lumbar MR images and corresponding medical records.

## Methods

### Subjects

Patients were not excluded from this project except the records were not in their integrity, or the MR image quality was poor, or the structure of spine was disordered, or other painful diseases, such as infection, trauma, spondylolisthesis, lumbar spinal stenosis syndrome, tumor, metabolic bone disease, were evidenced. If there were two or more records of one patient, the latest one was considered for inclusion in this study.

### MR imaging

Lumbar spine MR imaging scan was performed at imaging center using a 1.5 Tesla superconducting MR system (Signa, GE Medical System, Milwaukee, Wisconsin, USA). All patients examined in the supine position acquired sagittal T1-weighted spin echo (repetition time (TR): 450–500 msec; echo time (TE): 15–20 msec; 256 × 192 matrix; section thickness 3–4 mm; intersection gap 0.5–1 mm), sagittal T2-weighted spin echo (TR: 2200–3500 msec; TE: 90–120 msec; 256 × 192 matrix; section thickness 3–4 mm; intersection gap 0.5–1 mm), and axial T2-weighted spin echo (TR: 2400–3500 msec; TE: 90–120 msec; 256 × 256 matrix; section thickness 4 mm; intersection gap 0.5 mm) MR imaging with a fixed imaging protocol. The images were collected, transmitted, and stored in the picture archiving and communications system (PACS; GE Centricity, GE healthcare) or collected as printed film hard copies.

### CT discography

A standard posterolateral, extrapedicular approach using a single 22 gauge needle was used in each patient at L4–5 and above. L5-S1 discs were studied in the same approach except two lumbosacral discs were punctured transdurally. Needles were always introduced from the side opposite to any lateralizing HIZ to the center of the disc, guided by fluoroscopic imaging. Once needle tips were accurately placed, 1.5–3.0 ml of non-ionic contrast medium (Ultrovist 370, Schering, Berlin, Germany) was slowly injected into the nucleus. The injection was stopped if pain was induced, if firm resistance was encountered, or if contrast medium extended to the posterior edge of the vertebral body. X-ray films were obtained in the anteroposterior and lateral projections of each lumbar spine before needle removal. Post-discography CT scan was performed at the imaging center using a multi-slice spiral CT scanner system (Aquilion, Toshiba Medical Systems, Tokyo, Japan). In all CT scans, patients were in the supine position and settings remained at 120 kV, 250 mA, slice thickness of 2 mm, reconstruction interval of 2 mm, and reconstruction function of FC10.

### Outcome measures

Orthopedic doctors made their diagnosis of LBP after studying the patient’s symptoms. Criteria for HIZ were followed according to the original description of Aprill and Bogduk [3] and Wang et al. [15], i.e., a high-intensity signal located in the substance of the posterior or posterolateral annulus fibrosus, dissociated from the signal of the nucleus pulposus. Disc morphology was classified in modified Dallas discogram scale of grade 0 to 4 for annular disruptions, as described by Aprill and Bogduk [3] (Table 1). A disc was classified as no pain, atypical pain, or concordant pain according to the patient’s description during the provocative discography. The standard of positive diagnosis included both the patient’s concordant pain and one asymptomatic adjacent disc.

**Table 1:** Modified Dallas discogram scale

Grade	Morphology of CT discograms
0	Normal disc
1	Radial fissure involving the middle third of the annulus
2	Radial fissure extending to the middle third of the annulus
3	Radial fissure extending to the outer third of the annulus but involving < 30° of the disc circumference
4	Radial fissure extending to the outer third of the annulus and involving > 30° of the disc circumference

### Data collection

Several characteristics, including gender, age, body weight, and LBP symptom, were obtained from the medical record of the patients. The presence of annular HIZ on MR images was determined by two experienced radiologists in a blinded fashion, i.e., they were unaware of any demographic and clinical characteristics of the patients. The readings were independent and separate proceedings. Discography was performed by one experienced procedural discographer. During discography, the contrast medium is injected to pressurize the disc and the patient’s response to the injection is recorded. The biplanar post-discography radiographs and post-discography CT images were evaluated and rated blindly by two experienced radiologists. All data were entered into SPSS software for analysis.

### Statistical analysis

Measurement data were expressed as the mean, standard deviation (SD), and range. Enumeration data were expressed as proportions. Binary logistic regression analysis was employed to determine the association of gender, age, body weight, and LBP symptom with the presence of HIZ sign on lumbar spine MR images. The odds ratios (ORs) and their 95% confidence intervals (95%CI) were calculated for annular HIZ. Differences for ratios were compared using Pearson’s chi-square tests or Fisher’s exact test for categorical variables. For all of the statistical tests, *P* values less than 0.05 were considered statistically significant. Statistical analysis was performed using Statistical Package for Social Sciences (SPSS 15.0 for Windows, SPSS Inc., Chicago, IL).

## Results

Finally, the MR images and appropriate clinical records of six hundred and thirty-seven individuals (3185 discs) who underwent lumbar spine MR imaging between March 2010 and October 2015 were involved and reviewed. Among the subjects investigated, 201 exhibited HIZ sign on MR images, a prevalence per patient of 31.55%.

Of the 637 patients, 45.53% were women (*n* = 290) and 54.47% were men (*n* = 347). The incidences of HIZ on lumbar MR images in the male and female groups were 32.56% (113 patients) and 30.34% (88 patients) separately. There was no significant difference in the prevalence of HIZ between male and female (*χ*^2^ = 0.360, *P* = 0.548 > 0.05).

Patients ranged in age from 16 to 86 years (mean 49.4 years.; SD 16.0). Annular HIZ confirmed by two radiologists was present in patients of all age groups in the series. The incidence of HIZ on lumbar MR images in various age groups was listed in Table 2. The highest prevalence of HIZ disc was founded in 50–59 years group (38.03%), and 60–69 years group (37.93%) took second place. The lowest two prevalence of HIZ disc were in 20–29 years group (7.84%) and < 20 years group (11.54%).

**Table 2:** Incidence of HIZ on lumbar MR images in various age groups (*n* = 637)

Age group (years)	HIZ			Total
	Present	Absent	Incidence rate (%)	
< 20	3	23	11.54	26
20–29	4	47	7.84	51
30–39	28	63	30.77	91
40–49	55	99	35.71	154
50–59	54	88	38.03	142
60–69	33	54	37.93	87
70–79	20	53	27.40	73
> = 80	4	9	30.77	13

χ^2^ = 24.340, *P* = 0.001

In the sample group, body weight range was between 30 kg and 105 kg (mean 67.6 kg; SD 12.2). The incidence of HIZ on lumbar MR images in various body weight groups was summarized in Table 3. The general trend indicated that the incidence of HIZ increases with body weight growth. The prevalence was founded the lowest (6.45%) when the body weight is less than 50 kg. The risk of HIZ increases rapidly when the body weight is between 50 kg and 69.5 kg. In the 80–89.5 kg group, the HIZ incidence rate arrived at 38.54%, the highest in the study.

**Table 3:** Incidence of HIZ on lumbar MR images in various body weight groups (*n* = 637)

Body weight (kg)	HIZ			Total
	Present	Absent	Incidence rate (%)	
< 50	2	29	6.45	31
50–59.5	20	71	21.98	91
60–69.5	70	137	33.82	207
70–79.5	61	122	33.33	183
80–89.5	37	59	38.54	96
> = 90	11	18	37.93	29

*χ*^2^ = 16.384, *P* = 0.006

Three hundred and eighteen patients (49.92%) were diagnosed as LBP, whereas the other 319 patients (50.08%) were asymptomatic. Of the 318 symptomatic patients, 115 (36.16%) had HIZ. The asymptomatic group had HIZ discs in 86 (26.96%) of the 319 patients. It is proved that there is a significant difference in HIZ prevalence between symptomatic and asymptomatic patients (*χ*^2^ = 6.247, *P* = 0.012 < 0.05).

**Fig. 1:**
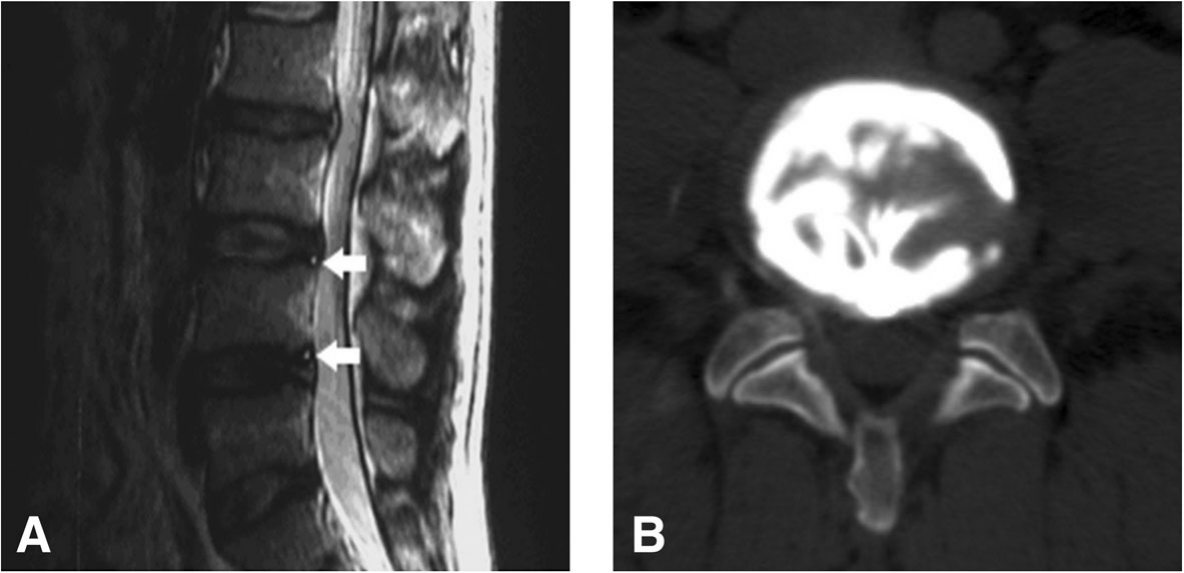
A 46-year-old man with low back pain for 9 months. **a** Sagittal T2-weighted MR image of the lumbar spine shows two HIZs at L3/4 and L4/5 (white arrow). **b** Axial post-discography CT image shows a grade 4 annular disruption at L3/4. Discography produced concordant pain

**Fig. 2:**
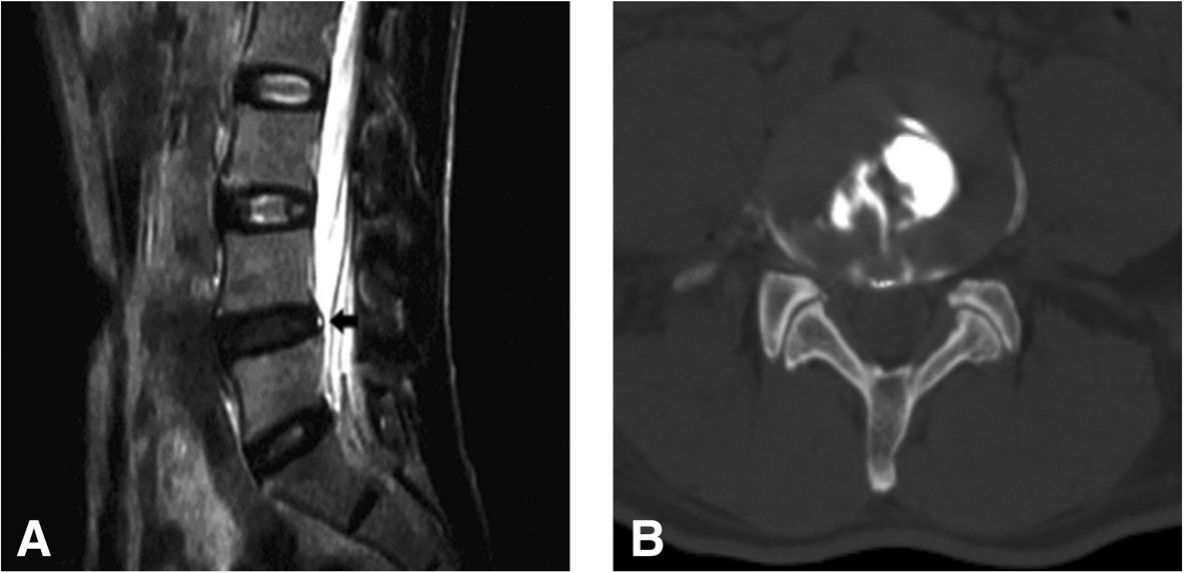
A 52-year-old man with low back pain for more than 1 year. **a** Sagittal T2-weighted MR image of the lumbar spine shows one HIZ at L4/5 (black arrow). **b** Axial post-discography CT image shows a grade 3 annular disruption at L4/5. Discography produced no pain

Discography and post-discography CT scanning were performed in a subset of 11 patients (16 HIZ discs). Patients ranged in age from 23 to 58 years (mean 45.3 years). These patients were referred for both MR imaging and later discography. Pain response according to the grade of annular disruption in 16 HIZ discs studied by CT discograms was reported in Table 4. All 16 of the HIZ discs exhibited grade 3 or above. Of the 9 painful and concordant HIZ discs, 8 exhibited grade 4 annular tears (Fig. 1), whereas 6 out of 7 non-concordant and non-painful HIZ discs exhibited only grade 3 annular tears (Fig. 2). The difference is proved statistically significant (*P* = 0.009 < 0.01).

**Table 4:** Pain response according to grade of annular disruption in 16 HIZ discs studied by CT discograms

Pain reproduction	Disruption grade^a^					Total
	0	1	2	3	4	
Positive	0	0	0	1	8	9
Negative	0	0	0	6	1	7
Total	0	0	0	7	9	16

^a^Modified Dallas discogram scale

^+^*P* = 0.009 (Fisher’s exact test)

Considering the clinical and statistical significance, the binary logistic regression model was constructed using four variables, i.e., gender, age, body weight, and LBP symptom, as shown in Table 5. Annular HIZ sign was significantly correlated with age (OR, 1.011; 95%CI, 1.001–1.023), body weight (OR, 1.022; 95%CI, 1.006–1.039), and LBP symptom (OR, 1.527; 95%CI, 1.083–2.152).

**Table 5:** Binary logistic regression analysis for lumbar disc HIZ on MR image

Variables	B	S.E.	Wald	OR	95% CI		*P*
					Lower	Upper	
Gender	0.012	0.191	0.004	1.012	0.696	1.472	0.948
Age	0.011	0.006	4.228	1.011	1.001	1.023	0.040^*^
Body weight	0.022	0.008	6.958	1.022	1.006	1.039	0.008^*^
LBP	0.423	0.175	5.834	1.527	1.083	2.152	0.016^*^

df = 1; ^*^ *P* < 0.05

## Discussion

Since the original definition of lumbar HIZ on MR images by Aprill et al. [3], many subsequent researchers reported the prevalence of HIZ in a wide range [7, 9, 12, 15, 16]. The latest cross-sectional study [16] involved a population-based cohort of 814 subjects and showed a prevalence of posterior HIZ as 21.9% (179/ 814). Another prospective observational study of 96 subjects [17] reported that the prevalence of HIZ in the total sample was 39.58%. In this study, the MR prevalence of HIZ arrived at 31.55%. The bias of sample source may be a likely reason for the difference in incidence.

Our study showed that there was no significant difference in the prevalence of HIZ between male and female, and a similar result was demonstrated by binary logistic regression analysis. Therefore, all subjects, regardless of gender, were merged into one sample in the subsequent analysis.

Boos et al. [18] analyzed histologic slices of 180 lumbar motion segment (44 individuals) and 30 surgical pathology specimens and founded that the first annular rim lesions with neovascularisation were detected before the age of 20 years. Then, during the third life decade, fissures in annulus fibrosus were observed, followed by cellular proliferation and blood vessels invasion along tears and clefts during the fourth decade and later. Peng et al. [5] harvested 11 surgical specimens of lumbar discs with HIZ for histologic examination and clarified that the HIZ lesion showed the vascularized granulation tissue in the tears of annulus fibrosus. They believed that the vascularized granulation tissue seems to be a product of healing reaction to annulus tear. In this study, HIZ was founded in all age group (from second decade to ninth decade). However, the prevalence of HIZ increased rapidly from the lower rates in the second and the third decades (11.54% and 7.84%) to the higher in the fourth decade (30.77%) and kept a slow increase during the following decades until the seventh decade. During the eighth decade and later, the HIZ incidence remained at approximately 30%. Above features of HIZ prevalence, on the whole, coincided with Boos’ suggestion on age-related changes in lumbar disc annulus fibrosus. Therefore, it was believed that the HIZ was an age-related sign on T2-weighted MR image of the lumbar spine.

Overweight has been proved a risk factor of spine disease [19]. Liuke et al. [20] performed a population-based follow-up MR imaging study and suggested that overweight increased the risk of lumbar disc degeneration. To our knowledge, overweight can lead to biomechanical changes in the lumbar spine, e.g., increasing the load of the lumbar disc. The effect of mechanical loads may alter the energy demand and nutrient supply and, hence, lead to disc degeneration and annulus fibrosus disruption [21]. Various mechanical stresses can induce apoptosis of nucleus pulposus cells and mediate intervertebral disc degeneration via the mitochondrial apoptotic pathway in nucleus pulposus cells in vivo [22]. Our result indicated that the body weight was positively associated with the HIZ prevalence (OR = 1.022), i.e., the heavier the body weight, the higher the incidence rate of HIZ. Therefore, it was suggested that body weight reduction within an appropriate range was an effective measure to reduce the risk of HIZ.

Low back pain, a common symptom in the general population, is described as one of the consequences of internal disc disruption by Crock [23]. After that, according to study the pathogenesis of discogenic LBP, Peng et al. [5] demonstrated that the presence of HIZ was a MR indication of annular fibrosus tears. These findings suggest some relationship between HIZ sign and LBP. Ross et al. [4] believed that granulation tissue or neovascularization is induced by inflammation. The tissue could produce some proinflammatory cytokine and mediators, e.g., prostaglandin E2, IL-6, and IL-8 [24], that can sensitize the nerve endings within the painful discs [17]. In this sample, the HIZ prevalence of symptomatic patients (36.16%) was significantly higher than that of asymptomatic (26.96%). Therefore, we believed that the association between lumbar annular HIZ on MR and LBP symptom was evidenced (OR = 1.527) in spite of the controversy on the diagnostic value of HIZ in LBP [5, 25].

Provocation discography has been clarified as a valuable method for the assessment of IDD and LBP [26]. Vanharanta et al. [27] reported 790 CT/discograms and discographic pain provocation in 291 patients and found that over 70% of fissures reaching the outer third of the annulus fibrosus (grade 3 disruption of Dallas discogram) were associated with pain reproduction. Aprill and Bogduk [3] reported 41 patients with a single HIZ and revealed either a grade 3 or a grade 4 annular disruption by CT/discogram. Our study showed that all the HIZ discs exhibited grade 3 or grade 4 disruptions, but only 9 discs (9/16) were detected with exact pain reproduction. Of the 9 painful and concordant HIZ discs, 8 exhibited grade 4 annular tears. This result reconfirmed the previous findings of Aprill and Bogduk [3] and demonstrated that the presence of HIZ was not an actual source of LBP, i.e., the association between HIZ and LBP might be indirect.

There were several potential limitations to our study. One limitation was the retrospective nature as a nonrandomized case-control study, which might contribute to bias in data processing. Another limitation was the shortage of subsample size. It was, therefore, suggested that a bigger subsample was needed for CT/ discogram in future studies. Finally, the study was limited by the data loss on body height. It was forced to choose the imperfect indicator of body weight instead of BMI.

## Conclusions

In conclusion, it is demonstrated that the presence of HIZ on lumbar MR image was associated with aging, high body weight, and low back pain symptom. HIZ sign indicated a part of the natural history of disc degeneration, but was not an actual source of low back pain.

## Abbreviations

95%CI: 95% confidence intervals
CT: Computed tomography
HIZ: High-intensity zone
IDD: Internal disc disruption
LBP: Low back pain
MR: Magnetic resonance
OR: Odds ratios
SD: Standard deviation
TE: Echo time
TR: Repetition time
